# Unveiling rootstock-induced dwarfing from comparative genomic analysis

**DOI:** 10.1186/s43897-024-00097-0

**Published:** 2024-05-06

**Authors:** Tingting Zhao, Quan Sun, Da-Gang Hu

**Affiliations:** National Research Center for Apple Engineering and Technology, Shandong Collaborative Innovation Center of Fruit & Vegetable Quality and Efficient Production, College of Horticultural Science and Engineering, Shandong Agricultural University, Tai’an, Shandong 271018 China

Since the 1950s and 1960s, the “green revolution” of cereal crops has provided new solutions to the food crisis faced by people around the world, and the core of the “green revolution” is the application of dwarfing genes (Pingali 2012). Therefore, the “green revolution” has also brought dwarfing traits into the vision of breeders worldwide. For woody and vine crops that typically undergo asexual reproduction, the use of dwarfing rootstocks is a more suitable approach to realize high-density planting and improve the economic benefits of orchards, which is unlike herbaceous crops such as grains (Boss and Thomas 2002; Mcclymont et al. 2021). Although previous studies have identified several quantitative trait locus (QTL) loci in apple rootstock that may induce dwarfing of the scion, the mechanisms underlying rootstock-induced dwarfing and the identification of crucial regulatory factors remains largely unrevealed (Foster et al. 2015; Wang et al. 2019). Moreover, the currently published apple (*Malus domestica* Borkh.) genomes mainly consist of cultivated varieties and a small number of wild relatives, such as ‘Gala’, GDDH13, HFTH1, Xinjiang Wild Apple (*Malus sieveisii*), etc. (Daccord et al. 2017; Sun et al. 2020). However, the lack of high-quality reference genome for apple rootstocks severely limits the exploration of mechanisms underlying rootstock-induced dwarfing. Therefore, deciphering the genome of apple dwarfing rootstocks is of great significance to explore the genetic mechanism of rootstock-induced dwarfing, achieve molecular design breeding, and promote modern reformation of apple planting and cultivation models (De Mori and Cipriani 2023).

Recently, in a paper published in Nature Genetics, Li et al. (2024) assemble the chromosome-level, near-gapless, and haplotype-resolved reference genomes of the popular dwarfing rootstock ‘M9’, the semi-vigorous rootstock ‘MM106’, and one of the most commonly grown apple cultivars ‘Fuji’. Further comparative analysis of their genomes indicates that the apple orthologue of auxin response factor 3 (MdARF3) is an important regulator inducing rootstock dwarfing. In addition, the authors develop a bioinformatics tool to accurately and effectively identify mobile mRNAs between rootstock and scion, and demonstrate these mobile mRNAs may also play significant roles in the architecture of dwarfing scion (Fig. 1).

**Fig. 1:**
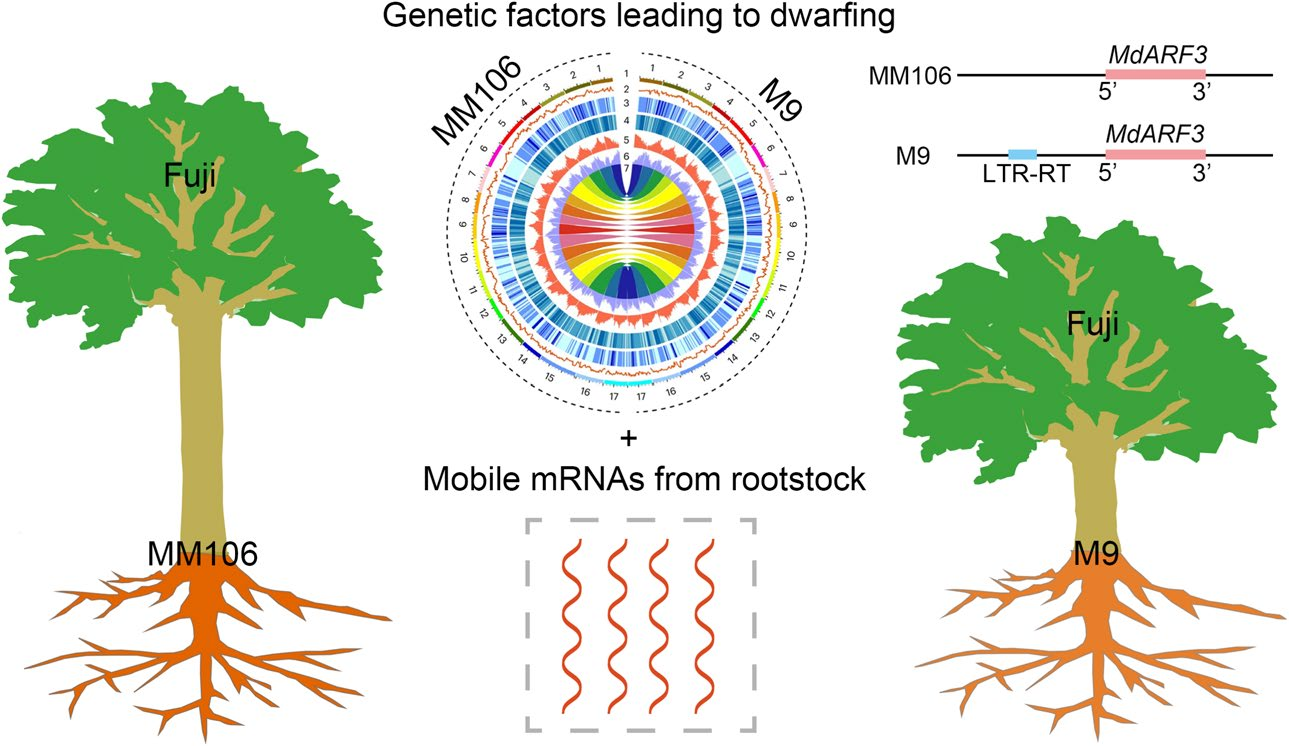
Genetic factors and mobile mRNAs from rootstock to scion are involved in rootstock-induced dwarfing

Since the East Malling Research Station in the UK cultivated the M series apple rootstock in the 1910s, multiple apple rootstock breeding institutions around the world have developed plentiful apple rootstock clones with diverse genetic relationships, such as the M and MM series from the UK, the R and CG series from the US, the B series from Soviet Union, and the “medium rootstock” and SH series from China. In order to clarify the phylogenetic relationship among different rootstock strains and elucidate the genetic background between rootstocks and wild and cultivated varieties, the authors collect asexual rootstock materials of apples worldwide, and construct an evolutionary tree, and find that remarkable introgression occured between wild and cultivated apple varieties during dwarfing rootstock breeding.

This research selects the most widely used dwarfing rootstock ‘M9’ and the semi-vigorous rootstock ‘MM106’, and assemblies a chromosome level, nearly gap free, and haplotype-resolved genomes of ‘M9’ and ‘MM106’ using deep HiFi and Hi-C sequencing. By comparing the previous reported QTL segment Dw1 with a higher contribution rate to rootstock-induced dwarfing, a 9,723-bp allele-specific long terminal repeat LTR-RT/gypsy insertion sequence is successfully identified in ‘M9’ and named DwTE. On the contrary, DwTE is absent in ‘MM106’. Interestingly, DwTE is located upstream an important factor in the auxin signaling pathway named MdARF3. Based on the relevance between gene expression difference in vigorous and dwarfing rootstocks, as well as the co-segregation of DwTE and dwarfing phenotype in vigorous and dwarfing populations, it is revealed that MdARF3 is probably a crucial regulator causing dwarfism in apple rootstocks. And the heterologous overexpression of *MdARF3* in Arabidopsis (*Arabidopsis thaliana*) shows longer internodes for the inflorescence compared to the control, providing further evidence for the role of MdARF3 in plant architecture (Liu et al. 2014). The role of MdARF3 in the dwarfism of apple rootstocks endows it with multiple potential application. For example, researchers can develop related molecular markers based on the difference of *MdARF3* upstream sequence, which is useful for the breeding or improvement of apple dwarfing rootstock and shorten breeding cycles. Moreover, breeders can create target germplasm resource by manipulating the expression level of *MdARF3* using gene editing methods.

The long-distance transport of mobile mRNAs between rootstock and scion also plays a significant role in regulating the traits of grafted plants (Liu et al. 2022), but it has always been a challenge to identify mobile mRNAs between rootstock and scion in apple. To address this problem, the authors develop a bioinformatics tool called “RNAGlass” that integrates genomic and transcriptome data to accurately and effectively identify mobile mRNAs between rootstock and scion based on the assembled high-quality reference genome of the cultivated variety “Fuji”. With the help of this tool, the mRNA transcripts transmitted between rootstock and scion during the critical period of dwarfing are identified, which strongly facilitates the comprehensive understanding of the molecular mechanism of rootstock dwarfing. On the other hand, the exploration of more potential rootstock dwarfing factors by this bioinformatics tool will provide useful sites for apple rootstock dwarfing breeding.

Taken together, this study successfully identifies the significant genes causing apple dwarfing, deciphers the genetic code behind apple rootstock dwarfism, and uncovers the mystery of apple dwarfism. Related results in this paper lay a solid foundation for the molecular design breeding of dwarfing rootstocks in the field of woody economic forests and fruits represented by apples, and will accelerate the fulfillment of “green revolution”.

## Data Availability

Not applicable.
